# Epithelial-mesenchymal transition in primary human bronchial epithelial cells is Smad-dependent and enhanced by fibronectin and TNF-α

**DOI:** 10.1186/1755-1536-3-2

**Published:** 2010-01-05

**Authors:** Joana Câmara, Gabor Jarai

**Affiliations:** 1 Novartis Institutes for BioMedical Research, Respiratory Disease Area, Wimblehurst Road, Horsham, RH12 5AB West Sussex, UK

## Abstract

**Background:**

Defective epithelial repair, excess fibroblasts and myofibroblasts, collagen overproduction and fibrosis occur in a number of respiratory diseases such as asthma, chronic obstructive pulmonary disease (COPD) and pulmonary fibrosis. Pathological conversion of epithelial cells into fibroblasts (epithelial-mesenchymal transition, EMT) has been proposed as a mechanism for the increased fibroblast numbers and has been demonstrated to occur in lung alveolar epithelial cells. Whether other airway cell types also have the capability to undergo EMT has been less explored so far. A better understanding of the full extent of EMT in airways, and the underlying mechanisms, can provide important insights into airway disease pathology and enable the development of new therapies. The main aim of this study was to test whether primary human bronchial epithelial cells are able to undergo EMT *in vitro *and to investigate the effect of various profibrotic factors in the process.

**Results:**

Our data demonstrate that primary human bronchial epithelial cells (HBECs) are able to undergo EMT in response to transforming growth factor-beta 1 (TGF-β1), as revealed by typical morphological alterations and EMT marker progression at the RNA level by real-time quantitative polymerase chain reaction and, at the protein level, by western blot. By using pharmacological inhibitors we show that this is a Smad-dependent mechanism and is independent of extracellular signal-related kinase pathway activation. Additional cytokines and growth factors such as tumour necrosis factor-alpha (TNF-α), interleukin-1 beta (IL1β) and connective tissue growth factor (CTGF) were also tested, alone or in combination with TGF-β1. TNF-α markedly enhances the effect of TGF-β1 on EMT, whereas IL1β shows only a very weak effect and CTGF has no significant effect. We have also found that cell-matrix contact, in particular to fibronectin, an ECM component upregulated in fibrotic lesions, potentiates EMT in both human alveolar epithelial cells and HBECs. Furthermore, we also show that the collagen discoidin domain receptor 1 (DDR1), generally expressed in epithelial cells, is downregulated during the EMT of bronchial epithelium whereas DDR2 is unaffected. Our results also suggest that bone morphogenetic protein-4 is likely to have a context dependent effect during the EMT of HBECs, being able to induce the expression of EMT markers and, at the same time, to inhibit TGF-β induced epithelial transdifferentiation.

**Conclusions:**

The results presented in this study provide additional insights into EMT, a potentially very important mechanism in fibrogenesis. We show that, in addition to alveolar epithelial type II cells, primary HBECs are also able to undergo EMT *in vitro *upon TGF-β1 stimulation via a primarily Smad 2/3 dependent mechanism. The effect of TGF-β1 is potentiated on fibronectin matrix and in the presence of TNF-α, representing a millieu reminiscent of fibrotic lesions. Our results can contribute to a better understanding of lung fibrosis and to the development of new therapeutic approaches.

## Background

Epithelial-mesenchymal transition (EMT) is the trans-differentiation of epithelial cells into mesenchymal cells. During this process, epithelial cells are removed of cellular polarity and epithelial cell-cell, as well as cell-matrix adhesion contacts are remodelled. Markers of polarized epithelial cells, such as E-cadherin and some cytokeratins, are lost whereas markers of mesenchymal cells such as vimentin, N-cadherin or of myofibroblasts, as α-smooth muscle actin (α-SMA) are acquired [1]. As a result of these changes, epithelial cells are converted into motile fibroblasts to myofibroblasts (FMT) that are key cells in both the degradation and *de novo *synthesis of the extracellular matrix (ECM). The trans-differentiated cells are able to migrate through basement membranes into other areas in the tissue. EMT has been shown to play a role in organogenesis during embryonic development and is an important biological process in normal wound healing [2]. However, disregulated EMT also appears to occur in disease, contributing to cancer progression and metastasis, as well as to the pathogenesis of chronic degenerative fibrotic disorders in several organs including the lung [3,4].

Dysfunction of the lungs and conducting airways at different levels may lead to a number of respiratory diseases such as asthma, cystic fibrosis, chronic obstructive pulmonary disease (COPD) and pulmonary fibrosis and an important role for EMT has been proposed in the pathogenesis of many of these [5-8]. Particularly, recent studies have identified important mechanisms that control the mesenchymal trans-differentiation of lung alveolar epithelial cells (AECs) in pulmonary fibrosis. It has been shown that both rat and human lung AECs can undergo EMT in response to exposure to transforming growth factor (TGF)-β1 in culture [9-11]. The EMT of alveolar cells was later demonstrated *in vivo *by generating transgenic mice expressing β-galactosidase exclusively in lung epithelial cells and following their fate after inducing lung fibrosis by intranasal instillation of adenoviral TGF-β1 [12]. Further evidence for the re-occurence of EMT in adult tissue, as well as for its possible role in pathogenesis, was obtained from lung tissue from idiopathic pulmonary fibrosis (IPF) patients by co-localization of myofibroblast and epithelial markers in AECs overlying fibroblastic foci, which are key features of the disease and are important for both diagnosis and disease progression [11].

A number of growth factors and cytokines are able to induce EMT *in vitro*. TGF-β is a central regulator of pulmonary inflammation and fibrosis [13]. TGF-β1 has been reported to induce EMT in AECs as well as the differentiation of fibroblasts to myofibroblasts and was also shown to be upregulated in IPF patients [11,14-16]. Several other factors have also been identified that appear to act in concert with or to modulate TGF-β activity. Whereas on its own IL (interleukin)1β does not induce EMT in A549 cells and similarly, tumour necrosis factor (TNF)-α has only a mild effect, when combined with TGF-β the two factors enhance EMT in type II AECs in both rat-derived cells and in the human A549 cell line. Similarly, connective tissue growth factor (CTGF) is produced by fibroblasts and bronchial epithelial cells and is also known to regulate fibroblast proliferation and collagen deposition in coordination with TGF-β [17,18].

The molecular mechanisms underlying EMT in the lung have not been explored in great detail. TGF-β1 is known to signal through two main pathways: the canonical Smad-dependent pathway and the alternative mitogen-activated protein kinase (MAPK) pathway [19]. Both of these have been implicated in EMT and FMT [7,20-22]. In addition, bone morphogenetic proteins (BMPs), members of the TGF-β superfamily, are involved in a variety of biological functions including lung morphogenesis [23]. The balance and interplay between BMP and TGF-β signalling has been demonstrated to be important to development [24]. Smad2 and Smad3 are activated by activin receptor-like kinase (ALK)-4, -5 and -7 receptors in response to TGF-β ligands, whereas Smad1, Smad5 and Smad8 are activated by ALK-1, -2, -3 and -6 in response to BMPs [25]. Several BMPs, particularly BMP7 and BMP4, have been implicated in EMT in various organs such as the kidney and lung [26,27].

The ECM also appears to be playing key roles in fibrosis not only as a major cause of the disruption of tissue architecture but also due to its ability to activate cellular pathways. The main ECM components of lung basement membranes are collagen IV and laminin [28]. For example, in IPF lung basement membranes are disrupted and the expression of collagen I, collagen III and fibronectin is upregulated [29-31]. Furthermore, fibronectin has been recently shown to drive the EMT of mouse AECII [12]. Two main families of receptors for ECM proteins have been implicated in lung cancer and fibrosis [32]: integrins and the discoidin domain receptors (DDRs). While integrins can sense various ECM proteins, DDRs appear to play a more specialized role. They are receptor tyrosine kinases that function as collagen receptors and have intrinsic signalling properties [33]. DDR1 is expressed primarily in epithelial tissues, whereas the other member of the subfamily, DDR2, is mostly present in mesenchymal cells [34]. The differential expression of DDR receptors appears to be context and disease dependent. Human nasopharyngeal carcinoma and HAK-1B hepatoma cells showed overexpression of DDR2, whereas DDR1 expression was unaltered [35,36]. In contrast, in human lung cancer tissue DDR1 was upregulated, whereas DDR2 expression was unaffected [37]. As collagen receptors, DDRs may play a role in mechanisms related to excessive collagen deposition, such as EMT or FMT in fibrosis. However, they have not yet been explored in this context.

Another mechanism that is proposed to contribute to the remodelling of diseases is the disturbed physiological balance between matrix formation and degradation. This equilibrium appears to be lost in fibrotic diseases and several matrix metalloproteases (MMPs), involved in both matrix turnover and growth factor activation, such as MMP-2 and MMP-9, have been shown to be upregulated [38]. These two MMPs also play a role in the cleavage and activation of the main profibrotic growth factor, TGF-β, and, hence, they may be of particular importance [39].

The identification of the specific epithelial cell types that are able to undergo EMT, and the underlying mechanisms, has recently been a matter of intense investigation as this may be an important process by which fibroblast numbers increase, constituting the fibroblastic foci correlated with severity of pulmonary fibrosis [40]. As described above, most of the work on lung EMT has focused on the alveolar epithelium. The increased expression of the mesenchymal marker S100A4 in airway epithelium biopsy samples from lung transplant patients suggests that the epithelium lining the conducting airways might also be able to differentiate into mesenchymal cells [41]. In the current study, we asked whether primary human bronchial epithelial cells, similarly to AECs, can also undergo EMT. We demonstrate that these cells can phenotypically convert to mesenchymal cells, an effect potentiated in a pathological environment reminiscent of fibrotic disease composed of TGF-β1 and fibronectin. Furthermore, we show that this is a Smad-dependent mechanism and implicate BMP4 and DDR1 in bronchial epithelial cell transdifferentiation.

## Materials and methods

### Cell culture

**Figure 1:**
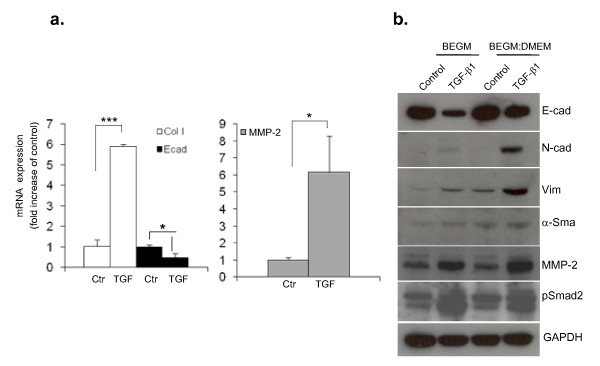
**Transforming growth factor (TGF)-β1 induces epithelial mesenchymal transition (EMT) in human bronchial epithelial cells (HBECs)**. HBECs were incubated with TGF-β1 (5 ng/ml) for 72 h in order to induce EMT. (a) Quantitative polymerase chain reaction analysis shows that TGF-β1 induces the mRNA expression of collagen I α 1 and matrix metalloprotease (MMP)2 and downregulates the epithelial marker E-cad. The relative expression level of each gene was normalized to glyceraldehyde 3-phosphate dehydrogenase (GAPDH) mRNA in the same sample. Statistical significance was determined by Student's *t *test; *P < 0.05, **P < 0.01, ***P < 0.001. (b) Western-blot analysis shows that TGF-β1 induces an upregulation of mesenchymal proteins (N-cad, vimentin, MMP-2) and of the myofibroblast protein α-smooth muscle actin and a downregulation of the epithelial marker E-cad in two different culture media. It also reveals an upregulation of phosho-Smad2 with TGF-β1 stimulation. An antibody against GAPDH was used as loading control. All further experiments were performed in the presence of BEGM medium unless indicated otherwise.

A549 cells (ATCC) were maintained in DMEM (Invitrogen, CA, USA) supplemented with 10% fetal bovine serum (FBS), 1% penicillin/streptomycin and human bronchial epithelial cells (HBECs; Lonza, Basel, Switzerland) were maintained in BEGM medium (Lonza), both at 37°C in the presence of 5% CO_2 _in a humidified incubator. Except for the experiments shown in Figure 1, all further experiments with HBECs were performed in BEGM medium only, unless indicated otherwise.

### EMT assay

A549 cells were seeded at a density of 6 × 10^4 ^cells/well (12-well plates) in DMEM 1% FBS and HBECs at a density of 10^6 ^cells/well in 1:100 BEGM:BEBM (six-well plates). Cells were allowed to adhere for 1 day and then changed into media containing 5 ng/ml of TGF-β1 (R&D Systems, MN, USA). A549 cells were differentiated for 48 h and HBECs for 3-5 days. Human BMP4 recombinant protein (5 or 50 ng/ml, R&D Systems) was used in some EMT experiments.

### EMT assay in the presence of pharmacological inhibitors

**Figure 2:**
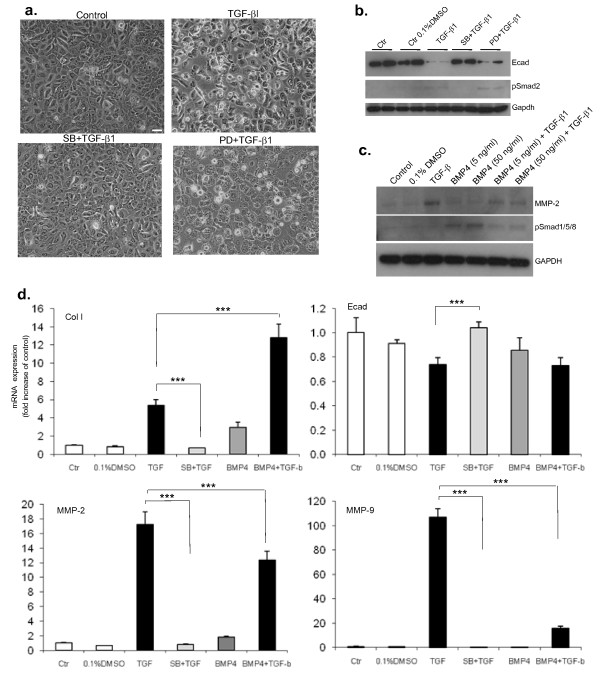
**Transforming growth factor (TGF)-β1-induced epithelial mesenchymal transition (EMT) in human bronchial epithelial cells (HBECs) is Smad-dependent**. HBECs that have been pre-incubated with the activin receptor-like kinase (ALK)-5 inhibitor SB431542 (SB, 10 μM) 1 h before the addition of TGF-β1 (5 ng/ml) are unable to undergo EMT after 5 days of differentiation. By contrast, mitogen-activated protein kinase inhibition by PD98059, a MEK inhibitor (PD, 10 μM), only partially inhibits EMT. (a) Representative phase contrast images (10× magnification, scale bar = 10 μM) show that HBECs in the presence of TGF-β1 lose cell-cell contact, become more sparse and change into an elongated fibroblastoid morphology. This effect is inhibited after pre-incubation of the cells with the ALK-4,-5,-7 inhibitor SB but not by the MEK inhibitor PD. (b) Western-blot analysis shows that SB completely abolishes TGF-β1 gene regulation of E-cad and the phosho-Smad2 signal is fully inhibited in the presence of SB. An antibody against glyceraldehyde 3-phosphate dehydrogenase (GAPDH) was used as a loading control. (c) Western-blot analysis shows that the upregulation of matrix metalloprotease (MMP)-2 by TGF-β1 is partially inhibited by the presence of bone morphogenetic proteins (BMP)4 confirming the quantitative polymerase chain reaction (qPCR) results shown in (d). Phosho-Smad1/5/8 shows active BMP signalling in the presence of BMP4 which is inhibited in the presence of TGF-β1. An antibody against GAPDH was used as loading control. (d) q(PCR) analysis shows that SB inhibits gene expression changes of collagen I α 1, MMP2, MMP-9 and E-cad induced by TGF-β1 (top 2 panels). BMP4 (50 ng/ml) induces a significant upregulation of collagen I in the presence of TGF-β1, as compared to TGF-β1 alone (top left panel) and has no significant effect on E-cad expression either alone or in combination with TGF-β1 (top right panel). In contrast, the upregulation of MMP-2 and MMP-9 expression in the presence of TGF-β1 is significantly inhibited by concomitant BMP4 treatment (bottom 2 panels). The relative expression level of each gene was normalized to GAPDH mRNA in the same sample. Statistical significance was determined by one-way ANOVA followed by Tukey test; ***P < 0.001.

**Figure 3:**
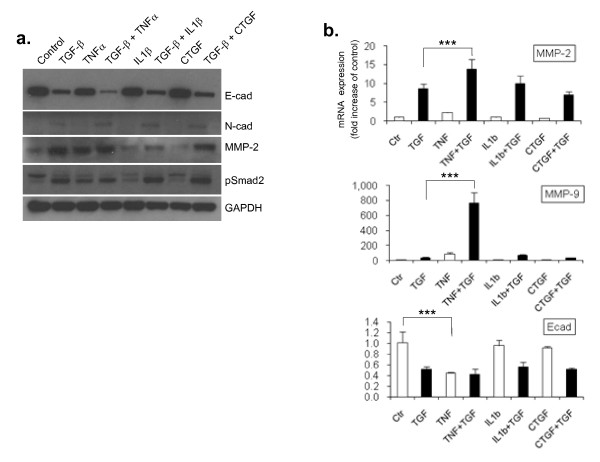
**Tumour necrosis factor (TNF)-α enhances the effect of transforming growth factor (TGF)-β1 on epithelial mesenchymal transition (EMT) induction in human bronchial epithelial cells (HBECs)**. (a) Western-blot analysis shows that TNF-α further potentiates the upregulation of N-cad and matrix metalloprotease (MMP)-2 and a downregulation of E-cad induced by TGF-β1. TNF-α, itself or in combination with TGF-β1 also induces Smad2 phosphorylation. An antibody against glyceraldehyde 3-phosphate dehydrogenase (GAPDH) was used as loading control. (b) Quantitative polymerase chain reaction analysis shows that TNF-α, when in combination with TGF-β1, induces a dramatic increase in MMP-2 and MMP-9 mRNA expression. Furthermore, TNF-α treatment alone reduces E-cad expression by approximately 50% whereas IL-1β or connective tissue growth factor have no effect on their own, neither do they potentiate TGF-β1 induced gene expression changes. The relative expression level of each gene was normalized to GAPDH mRNA in the same sample. Statistical significance was determined by one-way ANOVA followed by Tukey test; ***P < 0.001

**Figure 4:**
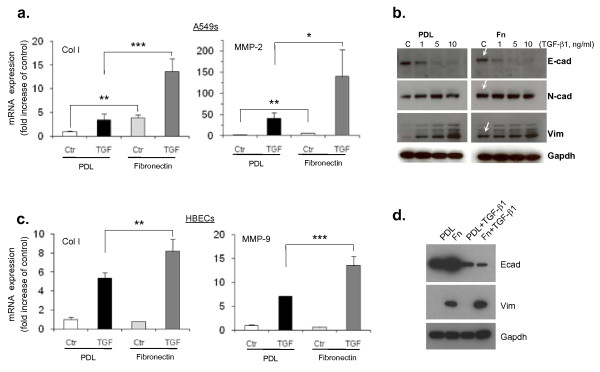
**Fibronectin promotes epithelial mesenchymal transition (EMT) in A549 cells and enhances transforming growth factor (TGF)-β1-induced EMT in human bronchial epithelial cells (HBECs)**. Human epithelial cells, either alveolar type II A549s (a and b) or primary human bronchial cells (c and d) were plated at equal cell density on PDL or fibronectin-coated dishes and EMT assays performed as described in Materials and Methods. (a and c) Quantitative polymerase chain reaction analysis shows a significant upregulation of collagen I and matrix metalloprotease-2 in A549 cells (a) and HBECs (c) grown on fibronectin compared to PDL in A. In the presence of TGF-β1, fibronectin shows a further significant upregulation of these EMT markers when compared to cells grown on PDL in both cell types. Statistical significance was determined by one-way ANOVA followed by Tukey test; *P < 0.05, **P < 0.01, ***P < 0.001. (b and d) Western-blot analysis shows upregulation of mesenchymal marker proteins and downregulation of epithelial marker proteins in cells on fibronectin as compared to control cells on PDL for both A549 cells (b) and HBECs (d). An antibody against GAPDH was used as loading control.

**Figure 5:**
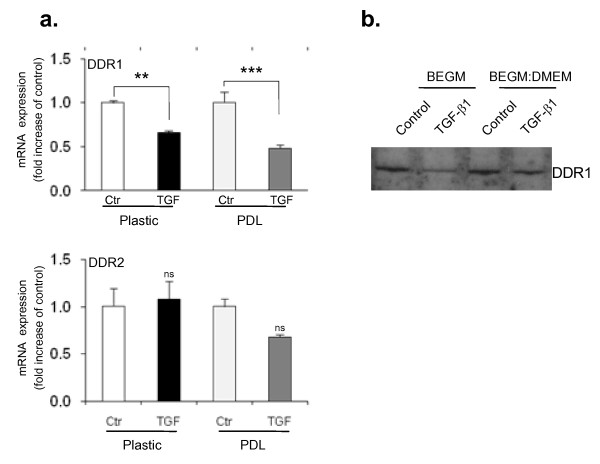
**Discoidin domain receptor (DDR)1 expression is downregulated in human bronchial epithelial cells (HBECs) following epithelial mesenchymal transition (EMT) whereas DDR2 expression is unaltered**. (a) Quantitative polymerase chain reaction analysis shows a 35% and 50% reduction of DDR1 expression after transforming growth factor (TGF)-β1 stimulation for 5 days in HBECs on plastic and PDL, respectively (top panel). However, DDR2 expression does not appear to change significantly in response to TGF-β1 treatment (bottom panel). The relative expression level of DDRs was normalized to glyceraldehyde 3-phosphate dehydrogenase mRNA in the same sample. Statistical significance was determined by one-way ANOVA followed by Tukey test; **P < 0.01, ***P < 0.001. (b) Western-blot analysis confirms a reduction of DDR1 protein expression level upon TGF-β1 treatment in HBECs in two different media.

A549 and HBECs were incubated with pharmacological inhibitors for 1 h prior to the addition of TGF-β1 (5 ng/ml) for EMT induction for 48 h or 3-5 days, respectively (see legends for Figures 1,2,3,4,5 for further description). The Smad pathway inhibitor SB431542 (10 μM, Sigma-Aldrich) and the ERK pathway inhibitor PD98059 (10 μM, Calbiochem) were used.

### Western blotting

Cells were lysed in a buffer containing 1% NP-40, 150 mM NaCl, 50 mM Tris pH 8.0, 1 mM sodium orthovanadate, 5 mM NaF and protease inhibitor cocktail (Sigma, NY, USA). Lysates were subjected to SDS-PAGE on a 4-10% Bis-Tris gels (NuPAGE, Invitrogen). They were then transferred onto nitrocellulose membranes (Invitrogen) and blocked in Tris-buffered saline (TBS), 0.1% Tween-20, 5% non-fat dried milk for 1 h at room temperature (RT). The blots were then washed three times in TBS 0.1% Tween (TBS-T) and incubated for 1 h at RT with a horseradish peroxidase-labelled secondary antibody (Invitrogen). After repeated washing in TBS-T the immunoreactive proteins were detected by chemiluminescence (ECL; Pierce, L, USA) according to the manufacturer's instructions. The following antibodies were used: E-cadherin (Abcam, MA, USA), N-cadherin (Zymed, CA, USA), vimentin (Abcam), α-SMA (Sigma), MMP-2 (R&D Systems), pSmad2 (Cell Signaling, MA, USA), pSmad1/5/8 (Cell Signaling), DDR1 (R&D Systems), glyceraldehyde 3-phosphate dehydrogenase (GAPDH; Santa Cruz Biotechnology, CA, USA).

### Real-time quantitative polymerase chain reaction (PCR)

Total RNA was prepared from cells using the RNeasy plus mini kit (Qiagen), according to manufacturer's instructions and RNA was reverse transcribedinto cDNA (Applied Biosystems, CA, USA). The cDNA was then amplified by real-time quantitative TaqMan PCR in a reaction containing 1× Taqman Universal PCR master mix, 1 μM primers, 0.3 μM probe and analysed using an ABI Prism 7900 sequence detector (Applied Biosystems). The house-keeping gene GAPDH was used as internal control. Data were normalized to GAPDH and expressed as fold change over control (Table 1).

**Table 1:** Sequences used for real-time quantitative polymerase chain reaction

Gene	Forward primer (5'-3')	Reverse primer (5'-3')	Probe
Collagen I	TGGCCTCGGAGGAAACTTT	TCCGGTTGATTTCTCATCATAGC	CCCCAGCTGTCTTAT

MMP-2	CGTCTGTCCCAGGATGACATC	TGTCAGGAGAGGCCCCATAG	6FAM-AGGGCATTCAGGAGC

MMP-9	TGGGCAGATTCCAAACCTTT	TCTTCCGAGTAGTTTTGGATCCA	6-FAM-CCACCACAACATCACCTA

E-cadherin	Pre-mixed primers and probe (Applied Biosystems, CA, USA)		

GAPDH	Pre-mixed primers and probe (Applied Biosystems)		

MMP, matrix metalloprotease; GAPDH, glyceraldehyde 3-phosphate dehydrogenase.

### Statistical analysis

The data show the mean ± standard deviation of at least three independent experiments (*n *= 3), unless otherwise indicated. Statistical significance was determined using one-way ANOVA or Student's *t *test. Statistical analysis was performed using GraphPad Prism software.

## Results

### TGF-β1 induces EMT in HBECs

In order to address the question of whether EMT can occur in AECs we first stimulated HBECs with TGF-β1 for 3-5 days. We observed a dramatic upregulation of collagen I mRNA expression and downregulation of the epithelial marker E-cad, as well as an enhanced expression of the metalloprotease MMP-2 after EMT induction (Figure 1a). Similarly, an increased expression of several mesenchymal markers including N-cad, vimentin, MMP-2 and of the myofibroblast marker α-SMA was detected at the protein level (Figure 1b). In contrast, a reduced expression of E-cad was observed in the presence of TGF-β1 (Figure 1b). We concluded from these data that TGF-β1 induces EMT in HBECs as revealed by a downregulation of epithelial markers and an upregulation of mesenchymal markers at both the RNA and the protein level. A concomitant upregulation of phospho-Smad2 with EMT induction in HBECs prompted us to investigate which signaling pathways were involved in this phenomenon.

### Smad inhibition prevents TGF-β1 induction of EMT in HBECs

In order to determine which of two main pathways, the Smad-dependent pathway or MAPK/ERK1/2 pathway - both of which can be activated by TGF-β, were involved in EMT in HBECs, we treated cells with pharmacological inhibitors for each pathway prior to the induction of EMT by TGF-β1. For the inhibition of the Smad pathway, we used SB431542, a potent and specific inhibitor of TGF-β type I receptor kinases (ALK-4,-5,-7), therefore an inhibitor of TGF-β signalling with no effect on ALK-1,-2,-3 and -6 and, hence, BMP signalling [42]. We found that treatment of HBECs with SB431542 before the addition of TGF-β1 prevented EMT, as demonstrated by the unchanged expression of marker proteins and their mRNA (Figure 2b and 2d) as well as by the preserved cellular morphology (Figure 2a). In contrast, the MEK/ERK inhibitor PD98059 failed to fully inhibit EMT (Figure 2a and 2b). Taken together, these results show that HBECs can undergo EMT *in vitro *in response to TGF-β1 via a primarily Smad-dependent mechanism.

### BMP4 is involved in EMT in HBECs

Two BMPs, BMP7 and BMP4, have so far been implicated in EMT in various organs. BMP7, and not BMP4, has been reported to reverse TGF-β1-induced EMT in the kidney and to induce mesenchymal to epithelial transition [27,43]. However, the evidence for the importance of BMP7 in the lung is less convincing, as BMP7 does not appear to inhibit EMT *in vitro *in A549s or *in vivo *in the bleomycin model [44]. Similarly, previous data on the role of BMP4 in EMT in the lung is rather conflicting. Whereas BMP4 was shown to induce EMT in the BEAS2B epithelial cell line, overexpressing Gremlin, an inhibitor of BMP4 signalling, also appeared to enhance EMT in A549 cells [26,45].

In order to determine whether BMP4 may also play a role in fibrosis pathogenesis of the airways via modulating EMT in HBECs, we incubated human BMP4 recombinant protein alone or before inducing EMT with TGF-β1 in HBECs. We found that BMP4 enhanced collagen I mRNA expression in the presence of TGF-β1 (Figure 2d). In contrast, the mRNA over expression of the metalloproteases MMP-2 and MMP-9 induced by TGF-β1 was inhibited by BMP4 (Figure 2d). An antagonistic effect of the BMP4 protein upon the TGF-β1-mediated upregulation of MMP2 protein was also confirmed by western blot (Figure 2c) and by assessing cellular morphology using phase contrast microscopy (data not shown). In order to assure that BMP signalling was active in the presence of BMP4 recombinant protein, we used an antibody that cross-reacts with the phosphorylated forms of Smads 1, 5 and 8, downstream effectors of BMP signalling. We found increased phosphorylation of Smad1/5/8 in the presence of both low (5 ng/ml) and high (50 ng/ml) concentrations of human BMP4 recombinant protein, an effect that could be abrogated if TGF-β1 was concomitantly added (Figure 2c). These results suggest a possible interplay between the BMP and TGFβ pathways during EMT in HBECs.

### TNF-α enhances the effect of TGF-β1 on EMT induction in HBECs

Next, we asked whether other cytokines and growth factors previously implicated in interstitial lung disease, such as TNF-α, IL1β and CTGF [46,47], alone or in combination with TGF-β1, would induce or alter the progression of EMT in HBECs. While TGF-β1 is involved in several biological processes and in EMT in many developmental and pathological scenarios, other mediators are likely to modulate TGF-β signalling conferring context specificity. Indeed, we found that TNF-α markedly enhanced the effect of TGF-β1 on EMT, whereas IL1β appeared to have a weak effect on MMP-9 expression but not on the expression of MMP-2 or Ecad (Figure 3a and 3b). Interestingly, CTGF, a profibrotic growth factor and a downstream effector of TGF-β signalling, had no significant effect when added alone or in combination with TGF-β1.

### Disease associated matrix enhances EMT in HBECs

In order to investigate if, in addition to soluble factors, cell-matrix contact would also modulate EMT in human airway epithelial cells we tested the effect of ECM components that have been associated with fibrotic lesions in IPF. Initially we tested whether type II A549 cells were affected by fibronectin and we found that alone it significantly upregulated mRNA expression of two important EMT markers, collagen I and MMP-2 (Figure 4a). Similarly, at the protein level, we detected significant induction of the mesenchymal markers N-cad and vimentin and the downregulation of the epithelial marker E-cad when cells were grown on fibronectin compared to cells grown on control plates coated with poly-D-lysine (PDL; Figure 4b).

We next looked to see if cellular contact with the fibronectin observed in AECII cells could also contribute to the progression of EMT in HBECs. In contrast to what we observed in alveolar type II epithelial cells, fibronectin on its own did not seem to have a significant effect on EMT in HBECs. Remarkably, however, when applied in combination with TGF-β1, fibronectin significantly enhanced TGF-β1-induced EMT in HBECs, as demonstrated by both the induction of mesenchymal and the downregulation of epithelial markers (Figure 4c and 4d).

### The collagen receptor DDR1 is downregulated in bronchial epithelial cells during EMT

Since the collagen receptors, DDRs, have been associated with remodelling and fibroproliferative diseases, we addressed the question of whether DDR expression was altered during EMT in bronchial epithelial cells. Although no DDR expression changes were detected in A549 epithelial cells during EMT (data not shown), DDR1 was consistently downregulated in HBECs at both the RNA and the protein level during TGF-β1-induced EMT (Figure 5a and 5b). In contrast, the other discoidin domain collagen receptor DDR2 expression did not show significant changes under any of the conditions tested.

## Discussion

The majority of the work on EMT in pulmonary fibrosis has so far been focused on AECs and it is less clear whether bronchial epithelial cells are able to undergo EMT. Further understanding of the EMT mechanisms in fibrosis is potentially of great importance as it could lead to approaches which impede or reverse the increase in the number of fibroblasts in fibrotic diseases.

Our work reveals that, in addition to AECII, primary HBECs are also able to convert to mesenchymal phenotypes in a Smad-dependent manner. While this work was in progress, other studies have recently also reported EMT in human airway epithelial cells. Zhang and colleagues have reported EMT-related changes in the polarized layer forming airway epithelial cell line 16HBE-14o [48] and our study extends these findings to primary HBECs. Borthwick and colleagues also support these findings [49]. In their study they demonstrated EMT of airway epithelial cells *in vitro *and mesenchymal cell marker expression in the epthelial cells in biopsies from obliterative bronchiolitis patients using immunohistochemistry. Furthermore, in a very recent study, airway epithelial cells isolated from asthmatics have been shown to be sensitized to TGF-β induced EMT [50]. These studies and our present work together provide supportive evidence for the occurence of EMT in airway epithelium in addition to the more extensively described process in the alveoli. Interestingly, it has been very recently reported that mouse tracheal epithelial cells can be induced to undergo EMT with TGF-β1 *in vitro *[51]. Whether this is the case with human tracheal cells remains to be determined. Nevertheless, a number of specialized airway and lung epithelial cell types appear to maintain an intrinsic plasticity that allows them to transdifferentiate into mesenchymal cells in pathological settings.

Our results also demonstrate that fibronectin alone is able to promote EMT in A549 epithelial cells and therefore extends the findings of Kim *et al*. [12] to the human setting, confirming an important role for fibronectin in the induction of EMT in human AECII. While fibronectin alone shows no significant effect in HBECs, EMT in these cells is potentiated in a pathological environment where fibronectin and excess TGF-β1 co-exist, further strengthening the relevance of our findings in disease.

Our study also gives new insight into some of the mechanism of EMT in bronchial epithelial cells. We show that DDR1 is downregulated during EMT in bronchial epithelium, whereas DDR2 is unaffected. Given that DDR1 is mostly expressed by epithelial, and not mesenchymal tissue, the reduction of its expression during EMT correlates with a decrease in epithelial identity. Remarkably, in a study comparing rat skin fetal fibroblasts of scar-free or scar-forming gestational ages, DDR1 was found to be downregulated at stages where scarring was no longer possible and collagen I was produced in excess. DDR2 levels were similar throughout gestation [52]. An analogy can be drawn with fibrosis in the adult where collagen I is overproduced and fibroblast numbers increase partly due to EMT and scarring of the tissue occurs. It therefore seems that, in situations of excess collagen and fibrosis, DDR1 can be downregulated in epithelial cells. Our findings also support recent reports on SLUG-induced EMT in Madin-Darby canine kidney cells with reduced DDR1 expression when induced to undergo EMT [53]. Shintani and colleagues (2008) [54] showed that collagen I-mediated upregulation of N-cadherin required cooperative signals from DDR1 and integrin β1 in pancreatic cells. Since we and others have shown that N-cadherin is upregulated in bronchial EMT, it will be interesting to evaluate in future studies whether integrins also play a role and whether there is any interplay with DDR1 in EMT in HBECs.

We have also investigated the effect of BMP4 during EMT of primary HBECs. Our data suggest a possible dual and context-dependent role for this growth factor. While it induced morphological EMT-like changes in HBECs, and was also able to upregulate collagen I expression, our data demonstrate that when applied in combination, BMP4 also inhibited TGF-β1-induced epithelial cell differentiation as demonstrated by its effect on MMP expression. Two epithelial cell lines were previously shown to have opposite EMT responses to BMP mediators. BEAS-2B, an airway epithelial cell line, was induced to undergo EMT when exposed to recombinant BMP4 [26,47]. On the other hand, the A549 AEC cell line overexpressing the BMP inhibitor Gremlin also showed enhanced EMT [26,47]. Furthermore, whereas Gremlin is over-expressed in the lungs of IPF patients and the responsiveness of IPF fibroblasts to BMP4 in culture is impaired, BMP4 and BMP7 activity is not reduced in IPF biopsies or in asbestos-exposed mouse lungs, as would be predicted by the increased expression of the BMP inhibitor Gremlin in IPF [47,55]. The BMP4 induced mechanisms thus appear to be complex as BMP4 activity can be modulated depending on the cell type and the cytokine millieu. It is interesting to speculate that the downregulation of MMPs, after BMP treatment, may not be a direct EMT effect but a paralell mechanism caused by the inhibitory effect of BMP signalling upon proteasome function [56]. In previous studies both MMP-2 and MMP-9 have been found to be downregulated by proteasome inhibition and the proteasome pathway has previously been linked to BMP signalling [56,57]. However, further studies are needed in order to unravel the complexities of BMP-TGFβ signalling interactions in EMT in bronchial epithelium.

The induction of the expression of MMPs, including MMP-2 and MMP-9 is well demonstrated in the context of cancer metastasis and is purported to play a role in both angiogenesis and cellular invasiveness [58] by contributing to the enhanced migratory phenotype of the cancer cell. Similarly, it was shown that, in *in vitro *injury models of repairing HBECs, MMP-9 accumulated in migrating HBECs at the leading edge of the wound [59]. Given the dramatic increase of MMP-9 expression that we have observed in HBECs undergoing EMT, it is reasonable to speculate that HBECs also gain a migratory phenotype during fibrosis pathogenesis in the lung. Our planned investigations will address this intriguing possibility in more detail.

Important recent advances demonstrate that several lung epithelial cell types can undergo EMT *in vitro *and suggest that the mechanism may also play a role *in vivo *during the development of various fibroproliferative diseases. Recent data from two groups using different detection methods demonstrated mesenchymal marker expression in airway epithelial cells of lung transplant patients with bronchiolitis obliterans [48,60]. Nevertheless, it is yet to be shown conclusively that EMT of bronchial epithelial cells plays a significant role in airway tissue remodelling and peri-bronchial fibrosis in the pathogenesis of lung diseases.

## Conclusions

Our results provide an additional insight into EMT, a potentially very important mechanism in fibrogenesis. We demonstrate that TGF-β1 can drive EMT in primary HBECs *in vitro*. We also show that this effect is primarily mediated via a Smad 2/3 dependent mechanism and is potentiated by cell-fibronectin interaction, in the presence of TNF-α, and can be further modulated by BMP pathway activation with BMP4. The results provided in this study establish the basis of future investigations into the mechanism of bronchial epithelial cell to mesenchymal cell differentiation, which, in turn, can contribute to a better understanding of lung fibrosis and to the development of new therapeutic approaches.

## Abbreviations

α-SMA: α-smooth muscle actin; ACK: activin receptor-like kinase; AECs: alveolar epithelial cells; BMP: bone morphogenetic proteins; COPD: chronic obstructive pulmonary disease; CTGF: connective tissue growth factor; DDR: discoidin domain receptor; ECM: extracellular matrix; EMT: epithelial mesenchymas transition; FBS: fetal bovine serum; FMT: fibroblasts to myofibroblasts transition; GAPDH: glyceraldehyde 3-phosphate dehydrogenase; HBEC: human bronchial epithelial cells; IL: interleukin; IPF: idiopathic pulmonary fibrosis; MAPK: mitogen-activated protein kinase; MMP: matrix metalloprotease; PCR: polymerase chain reaction; PDL: Poly-D-Lysine; RT: room temperature; SDS-PAGE: sodium dodecyl sulphate polyacrylamide gel electrophoresis; TGF: transforming growth factor; TNF: tumour necrosis factor; TBS: Tris-buffered saline; TBST: TBS tween.

## Competing interests

The authors declare that they have no competing interests.

## Authors' contributions

JC carried out the experiments and data analysis. GJ and JC designed the experiments, interpreted the data and wrote the final manuscript.
